# Thermal Exfoliation and Phosphorus Doping in Graphitic Carbon Nitride for Efficient Photocatalytic Hydrogen Production

**DOI:** 10.3390/molecules29153666

**Published:** 2024-08-02

**Authors:** Lu Chen, Linzhu Zhang, Yuzhou Xia, Renkun Huang, Ruowen Liang, Guiyang Yan, Xuxu Wang

**Affiliations:** 1 Fujian Province University Key Laboratory of Green Energy and Environment Catalysis, Ningde Normal University, Ningde 352100, China; llzhanglinzhu@163.com (L.Z.); yzxia@ndnu.edu.cn (Y.X.); t1432@ndnu.edu.cn (R.H.); t1629@ndnu.edu.cn (R.L.); 2 State Key Laboratory of Photocatalysis on Energy and Environment, Fuzhou University, Fuzhou 350002, China

**Keywords:** graphitic carbon nitride, P doped, hydrogen evolution, thermal exfoliation

## Abstract

Photocatalytic H_2_ evolution has been regarded as a promising technology to alleviate the energy crisis. Designing graphitic carbon nitride materials with a large surface area, short diffusion paths for electrons, and more exposed reactive sites are beneficial for hydrogen evolution. In this study, a facile method was proposed to dope P into a graphitic carbon nitride framework by calcining melamine with 2-aminoethylphosphonic acid. Meanwhile, PCN nanosheets (PCNSs) were obtained through a thermal exfoliation strategy. Under visible light, the PCNS sample displayed a hydrogen evolution rate of 700 μmol·g^−1^·h^−1^, which was 43.8-fold higher than that of pure g-C_3_N_4_. In addition, the PCNS photocatalyst also displayed good photostability for four consecutive cycles, with a total reaction time of 12 h. Its outstanding photocatalytic performance was attributed to the higher surface area exposing more reactive sites and the enlarged band edge for photoreduction potentials. This work provides a facile strategy to regulate catalytic structures, which may attract great research interest in the field of catalysis.

## 1. Introduction

Solar energy is regarded as the most promising candidate for renewable green energy to avoid environmental pollution and address the global severe energy crisis [1,2]. As an attractive strategy to utilize solar energy, photocatalytic water splitting has been considered a sustainable method for generating clean hydrogen [3,4]. To date, graphitic carbon nitride has been regarded as a promising semiconductor photocatalytic material in the field of photocatalysis. Importantly, graphitic carbon nitride possesses an excellent electronic structure, outstanding physiochemical properties, and a suitable band gap (2.7 eV) [5,6]. Therefore, graphitic carbon nitride has gradually become a competitive nano-semiconductor material for hydrogen energy production.

Nevertheless, pristine g-C_3_N_4_ suffers from several drawbacks, including poor electric conductivity, a low specific surface area, and the rapid recombination of photogenerated electron–hole pairs [7]. These drawbacks result in the inferior photocatalytic activity of g-C_3_N_4_. To address these drawbacks, several kinds of strategies have been developed to engineer the chemical composition and structure of g-C_3_N_4_, for example, cocatalyst loading [8,9], elemental doping [10,11], controlling its morphology [12,13], the construction of heterojunctions with other semiconductors [14,15], molecule incorporation [16], defect engineering [17,18], and nanostructure design [19]. Among these, element doping offers an effective strategy to regulate its electronic structure and extend its light absorption, further enhancing the photocatalytic activity of g-C_3_N_4_. Currently, non-metal elements (Br, B, S, O, and P) with different electronegativities can be doped into the g-C_3_N_4_ framework to tune the band gap structure, further accelerating the photogenerated carriers’ separation [20]. Phosphorus is the most earth-abundant non-metal element rich in electrons that can serve as an electron donor. Chen et al. reported doping P into a g-C_3_N_4_ framework to form P–N bands, which can accelerate the charge separation and transfer efficiency and further improve the hydrogen evolution [21]. P doping into g-C_3_N_4_ (PCN) has achieved remarkable enhancements of its photocatalytic activities, but it still fails to satisfy the practical requirement of synthesis tuning the band gap structure of the photocatalyst. Therefore, it is urgent to seek a new method to prepare a large-surface area photocatalyst to create more reactive sites and further enhance the photocatalytic activity of g-C_3_N_4_.

A two-dimensional (2D) g-C_3_N_4_ nanosheet exhibits a large specific surface area, more reactive sites, a short charge transfer distance, and quantum effects. Various strategies have been developed to prepare large-specific surface area g-C_3_N_4_ nanosheets, such as water steam exfoliation, sonification exfoliation, and ball milling. However, these strategies use solvents for the exfoliation process, which is time-consuming and inefficient. Qiu et al. reported a thermal exfoliation method to prepare single- or few-layered nanosheets with strengthened surfaces and semiconductor properties. Therefore, combining element doping and a thermal exfoliation method to prepare g-C_3_N_4_ with a large specific surface area improves its photocatalytic performance.

In this work, we successfully developed a green and facile method to prepare PCNSs with a large specific surface area. As expected, thermal exfoliation could effectively exfoliate the bulk PCN into few-layered nanosheets. The PCNS sample exhibited the highest photocatalytic hydrogen evolution rate of 700 μmol·g^−1^·h^−1^, which was 43.8-fold higher than that of g-C_3_N_4_. Its apparent quantum efficiency reached 0.49% under visible light of 420 nm. Meanwhile, the photocatalyst was subjected to four successive cycles and exhibited excellent photostability. This outstanding photocatalytic performance was attributed to its large surface area, which could afford more reactive sites for hydrogen evolution. Meanwhile, the introduction of P–N bonds could accelerate the charge carrier separation and transfer efficiency, leading to more efficient photocatalytic hydrogen production. This work paves a new way to construct high-surface area PCNSs by integrating element doping and thermal exfoliation.

## 2. Results

**Figure 1:**
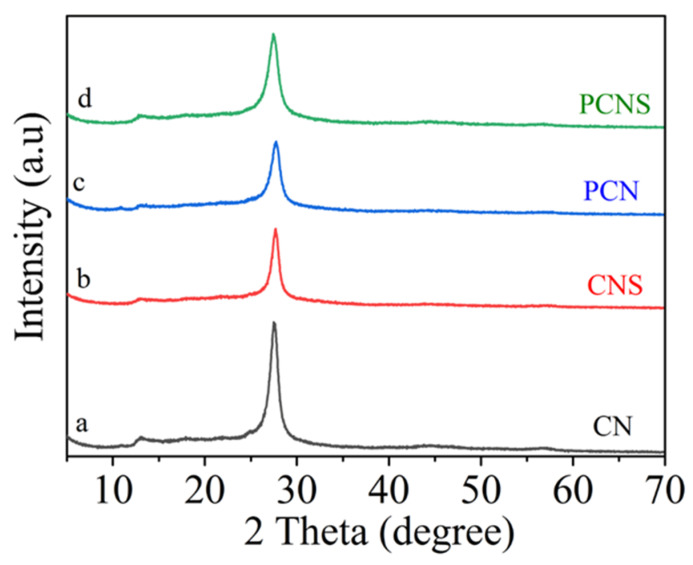
XRD patterns of the four photocatalysts.

The synthesized materials were investigated by X-ray diffraction (XRD) to measure their crystal phases and compositions. As shown in Figure 1a, the diffraction peaks located at 13° and 27° were ascribed to the (100) and (002) crystal planes [22], respectively. The former (100) plane was ascribed to the in-plane structure stacking pattern [23], while the latter (002) plane corresponded to the interlayer stacking [24,25]. Through the thermal exfoliation reaction process, the CNS photocatalyst maintained the original structure of the bulk g-C_3_N_4_ (Figure 1b). Compared with g-C_3_N_4_, the intensities of the two peaks of PCN were reduced. The diffraction peak in the (002) plane was slightly shifted to a small angle (Figure 1c). The reduction in the diffraction angle revealed the increased (002) interplane distance, which was ascribed to the radius of P being much bigger than that of C or N. As presented in Figure 1d, the PCNS sample maintained the original structure of the bulk g-C_3_N_4_. Meanwhile, the characteristic peak intensity became weaker and broader.

**Figure 2:**
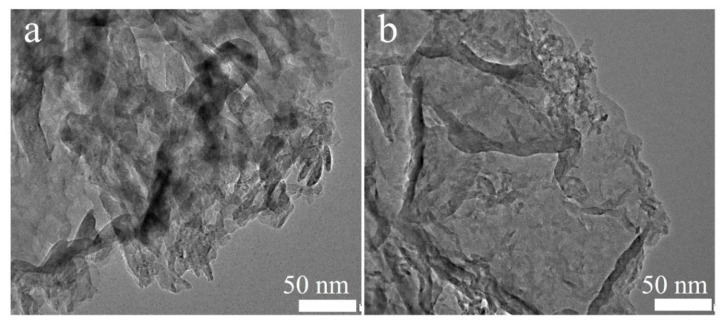
TEM images of (**a**) CN and (**b**) PCNS.

The morphologies and microscopic structures of all the samples were examined by SEM and TEM. As shown in Figure 2a, the pristine g-C_3_N_4_ consisted of two-dimensional nanosheets with curling edges, which was attributed to the multilayer structure of the graphitic properties of carbon nitride. As presented in Figure S1a, the CNS maintained the two-dimensional nanosheet structure after the thermal exfoliation reaction process. After doping with the P element, the SEM image of PCN exhibited nearly no changes, revealing that the structure remained intact after the elemental doping (Figure S1b). Moreover, the PCNS sample still retained the two-dimensional structure after the thermal exfoliation reaction process (Figure 2b). In addition, the PCNS’s specific surface area reached 76.99 m^2^·g^−1^, which was 8, 1.35, and 9.4 times higher than that of PCN, CNS, and g-C_3_N_4_ (Figure S2). The above results further confirm that thermal exfoliation can enlarge the surface area, afford more reactive sites, and reduce the carriers’ diffusion distance, further improving the photocatalytic performance of g-C_3_N_4_.

**Figure 3:**
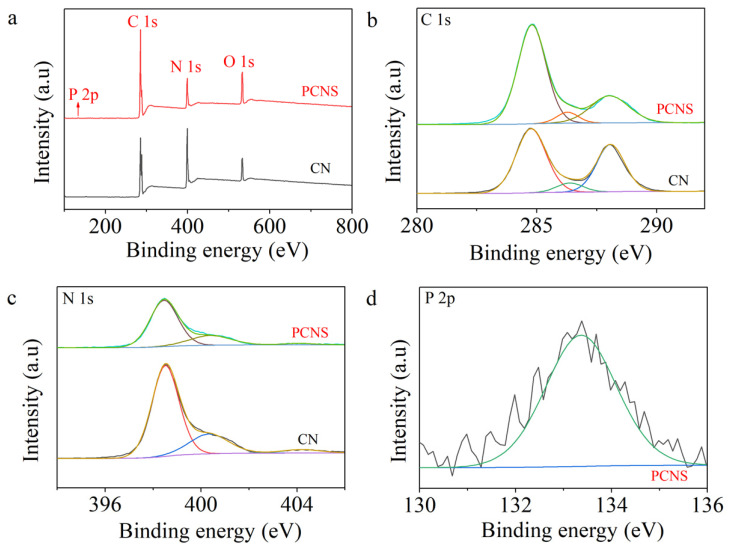
(**a**) XPS survey spectra. High-resolution spectra for (**b**) C 1s, (**c**) N 1s, and (**d**) P 2p.

The chemical states and compositions of g-C_3_N_4_ and the PCNS were studied by XPS. The XPS survey spectra in Figure 3a reveal that the two samples contained C and N elements. Moreover, the element of P was observed in the PCNS. As presented in Figure 3b, it can be observed that g-C_3_N_4_ exhibited three typical C 1s peaks located at 284.78, 286.38, and 288.08 eV, attributed to the physically absorbed carbon species or sp^2^ C–C bonds, C–NH_2_ species, and sp^2^–bond carbon (N–C=N) in the g-C_3_N_4_ aromatic ring [26]. The high-resolution spectrum of N 1s in g-C_3_N_4_ was divided into three peaks located at 398.48, 400.38, and 404.28, eV. The peak centered at 398.48 eV was attributed to the sp^2^-hybridized nitrogen (C–N=C group) [27,28]. The peak located at 400.38 eV was assigned to the tertiary N (C_3_–N or C_2_–N–H) [29,30]. The peak at 404.28 eV was related to the amino functional group (C–N–H) [8,31,32,33]. Compared with pristine g-C_3_N_4_, the C 1s and N 1s spectra of the PCNS were downshifted to low binding energies, which was ascribed to the P-doped change in the surface charge distribution. In addition, the characteristic peak of P 2p at 133.38 eV corresponded to the P–N bond in the PCNS [34,35,36,37].

**Figure 4:**
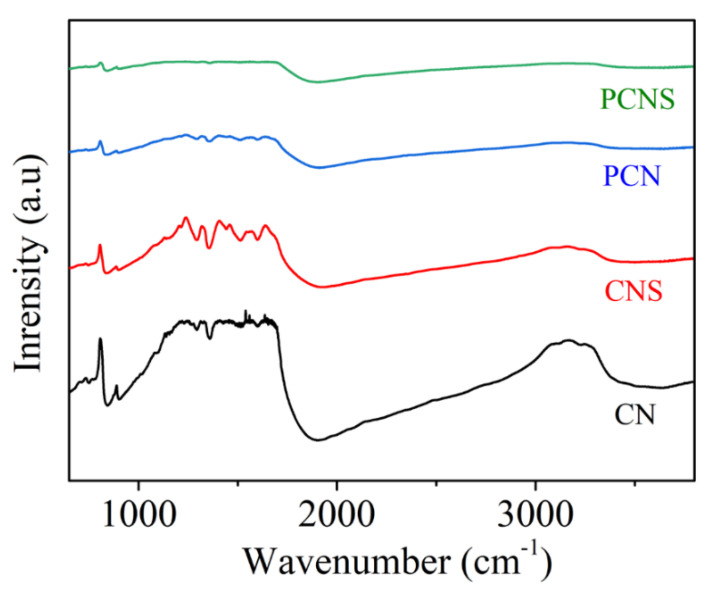
FTIR spectra of four photocatalysts.

To gain insight into their molecular structures, all the photocatalysts were investigated by FTIR. As presented in Figure 4, the peak located at 810 cm^−1^ was assigned to the out-of-plane bending mode of the heptazine units [38]. The peak in the range of 880–1640 cm^−1^ could correspond to the N–C=N heteroaromatic rings [39,40,41]. A characteristic peak for the aromatic C–N and C=N stretching vibrational model was shown at 1200–1700 cm^−1^ [42]. The peak centered at 3160–3440 cm^−1^ was attributed to the N–H stretching vibrations [43,44,45]. After the P-doping and thermal exfoliation reaction processes, the CNS, PCN, and PCNS photocatalysts maintained the original molecular structure of g-C_3_N_4_.

**Figure 5:**
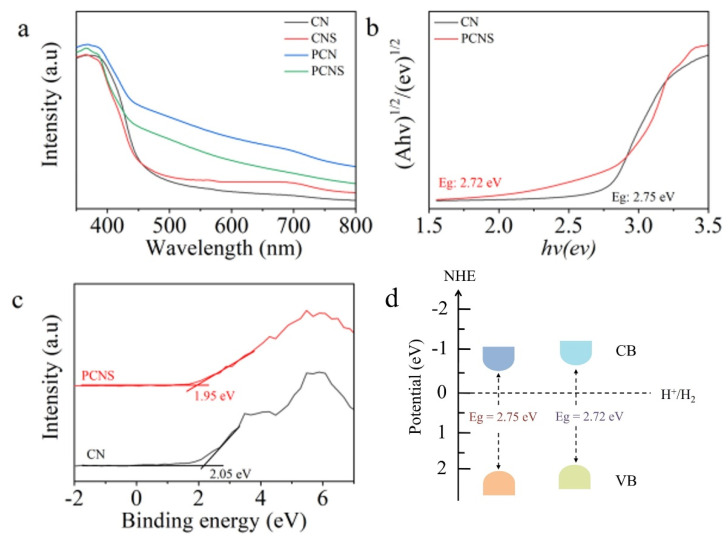
Optical and electronic properties of CN and PCNS. (**a**) UV–vis diffuse reflectance spectra, (**b**) plots of the transformed Kubelka–Munk functions versus photon energy, (**c**) VB-XPS, and (**d**) CB and VB positions.

The optical properties of all samples were measured by UV–vis absorption spectroscopy. As displayed in Figure 5a, the absorption edge of g-C_3_N_4_ was mainly located at 460 nm. In comparison, the absorption of CNS exhibited an obvious blueshift, which was ascribed to the quantum size effect. After P doping, the PCN sample absorption band edge had a minor redshift, expanding to the visible-light region. After the thermal exfoliation reaction process, the PCNS featured a blueshift, which corresponded to the quantum size effect. In addition, according to the Tauc method, the band gaps of g-C_3_N_4_ and the PCNS were 2.72 and 2.75 eV, respectively. As presented in Figure 5c, the VB potentials of g-C_3_N_4_ and the PCNS were 2.05 and 1.95 eV, respectively. Thus, the VB values of g-C_3_N_4_ and the PCNS were calculated to be 2.15 and 2.05 eV, respectively, according to the following equation:

E_VB-NHE_ = Ψ + E_VB-XPS_ − 4.44 (1)

Ψ: the electron work function of the XPS analyzer; E_VB-XPS_: the VB value tested by the VB-XPS plots; and E_VB-NHE_: the standard hydrogen electrode potential. Then, the valence band position was measured by the following equation:

E_CB_ = E_VB_ − Eg (2)

The results exhibit that the E_VB_ values of g-C_3_N_4_ and the PCNS were −0.57 and −0.7 eV, respectively. The energy band positions of g-C_3_N_4_ and the PCNS are schematically displayed in Figure 5d. The shifted CB and VB position of the PCNS led to a larger thermodynamic driving force for photocatalytic redox reactions.

**Figure 6:**
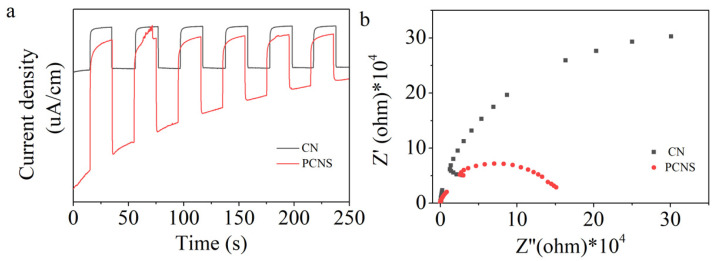
(**a**) Photocurrent and (**b**) EIS plots of CN and PCNS.

Photoelectrochemical analysis was conducted to gain further insight into the charge separation and transfer efficiency. Figure 6a presents the periodic on/off photocurrent responses of the CN and PCNS electrodes at 0.2 V (vs. Ag/AgCl) with a 1.0 M Na_2_SO_4_ electrolyte under visible-light illumination (λ > 420 nm). The photocurrent density of the PCNS was higher than that of CN, indicating the fast separation and transfer efficiency of the photogenerated electron–hole pairs after the P-doping and thermal exfoliation strategies.

Meanwhile, the electrochemical impedance spectra of the CN and PCNS electrodes using 10 mM K_3_[Fe(CN)_6_]/K_4_[Fe(CN)_6_] as the electrolyte in the dark are shown in Figure 6b. The PCNS shows a smaller arc radius than pristine CN, revealing its lower charge transfer resistance and fast charge separation (Figure 6b). Subsequently, the CN sample shows a strong PL emission peak intensity. The PL intensity of the PCNS significantly decreased, confirming that the recombination of photogenerated electron–hole pairs was effectively suppressed (Figure S3). Based on the above results, the P-doping and thermal exfoliation reaction processes can promote the separation and transfer efficiency of photogenerated hole–electron pairs.

**Figure 7:**
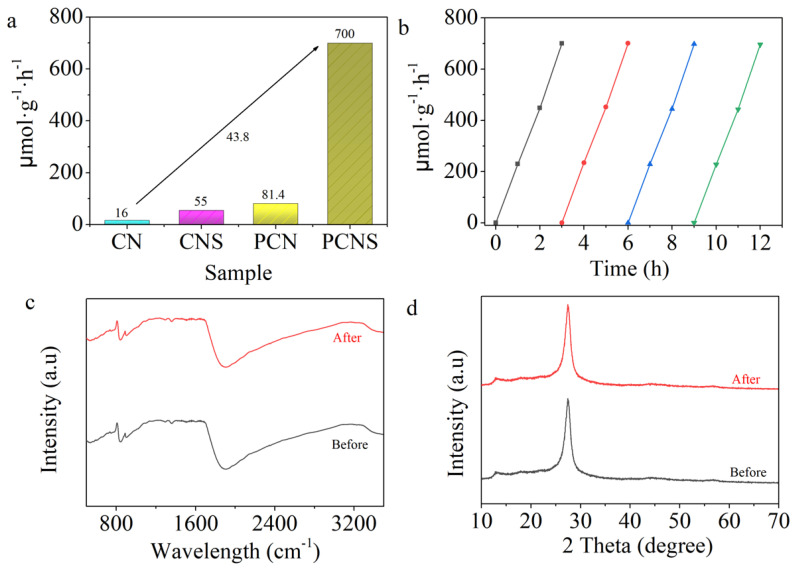
Photocatalytic performances of (**a**) CN, CNS, PCN, and PCNS; (**b**) cycling experiments of PCNS; and (**c**) FTIR and (**d**) XRD patterns of PCNS before and after reaction.

The photocatalytic activity of all samples was tested under visible-light irradiation using 100 mL of an aqueous solution containing 10% TEOA as a sacrificial agent. No hydrogen gas was detected without irradiation or a photocatalyst. As depicted in Figure 7a, the hydrogen evolution rate of pristine g-C_3_N_4_ was negligible (16 μmol·g^−1^·h^−1^). Then, after the thermal exfoliation reaction process, the CNS exhibited a high value of 55 μmol·g^−1^·h^−1^. After P doping, PCN showed that its photocatalytic performance was 81.4 μmol·g^−1^·h^−1^, which was 5.1 and 1.5 times higher than that of g-C_3_N_4_ and CNS. Meanwhile, the PCNS exhibited a high photocatalytic activity of 700 μmol·g^−1^·h^−1^, which was 43.8 times higher than that of CN. To further prove that the P-doping and thermal exfoliation reaction processes can enhance the photocatalytic performance of g-C_3_N_4_, comparisons between the photocatalytic abilities of the photocatalysts in the related reports and the as-prepared photocatalysts are listed in Table S1. Our work exhibited an improved H_2_ evolution rate compared with other reported photocatalysts. The remarkable photocatalytic performance of the PCNS was ascribed to its higher surface area affording more reactive sites. In addition, the AQE reached 0.49% under 420 nm light irradiation for the PCNS. Photostability is an important parameter for a photocatalyst’s application. As depicted in Figure 7b, the H_2_ evolution rate almost kept the same value during the four-cycle reaction, revealing excellent photocatalytic stability. Moreover, the XRD and FTIR patterns of the PCNS before and after the reaction did not change, confirming the favorable stability of the PCNS (Figure 7c,d).

## 3. Materials and Methods

### 3.1. Materials

Melamine, H_2_PtCl_6_·6H_2_O (37.5 wt% Pt), and 2-aminoethylphosphonic acid (AEP) were purchased from Sinopharm Chemical Reagent Co., Ltd. (Shanghai, China).

### 3.2. Preparation of g-C_3_N_4_

An amount of 10 g of melamine was placed into an alumina crucible with a cover. Then, the powder was calcined at 550 °C for 4 h with a ramp rate of 5 °C/min in a muffle furnace under an Ar atmosphere (50 mL/min). After cooling to room temperature, the obtained sample was denoted as g-C_3_N_4_ (CN).

### 3.3. Preparation of PCN

In a typical process, 10 g of melamine was added to 30 mL of deionized water. Next, 0.28 g of 2-aminoethylphosphonic acid was added to the mixed solution. Then, the mixed solution was kept at 100 °C under magnetic stirring at 100 rpm overnight. Finally, the obtained powder was calcined at 550 °C for 4 h under an Ar atmosphere (50 mL/min). The obtained brown powder was denoted as PCN.

### 3.4. Preparation of PCNS

An amount of 600 mg of PCN was placed into a porcelain boat and then sent into a tube furnace and calcined at 550 °C for 4 h under an Ar atmosphere (50 mL/min). After cooling to room temperature, the obtained powder was labeled as PCNS. The CNS sample was obtained through the same method.

### 3.5. Characterizations

The crystal structures and phase compositions of the photocatalysts were recorded by a Bruker XRD advance X-ray diffractometer (Salbruken, Germany) system using a Cu Kα X-ray source (λ = 0.15406 nm). The morphologies and microstructures were characterized by SEM (FE-SEM, JSM-6701F, JEOL, Tokyo, Japan ) and TEM (Tecnai Model G2 F20 S-TWIN, Peabody, MA, USA). The chemical states of the elements were analyzed by XPS (XPS, VG ESCALAB 250, Thermo Fisher Scientific, East. Grinstead, UK) with 150 W Al Ka X-ray radiation. C 1s (284.8 eV) was calibrated as the binding energy. The UV–vis diffusion spectra of all the photocatalysts were obtained with a Cary500 spectrophotometer with BaSO_4_ powder as the reflectance standard in the range of 800–4000 cm^−1^. The photoluminescence spectra (PL) were measured with a fluorophotometer (Edinburgh FL/FS900, Livingston, Scotland, UK) with an excitation wavelength of 400 nm. The functional groups of all samples were characterized by the FTIR spectra using a Nicolet 670 (Salbruken, Germany). The nitrogen adsorption–desorption isotherms were obtained using a nitrogen adsorber (ASAP 2020, Norcross, GA, USA). The photoelectrochemical values were measured in a standard three-electrode system using a CHI660E electrochemical workstation (CHI-660, Shanghai, China). A Pt wire and Ag/AgCl were the counter electrode and reference electrode. The working electrode was prepared by dropping 10 uL of a 5 mg/mL photocatalyst suspension onto the conductive surface of ITO glass and then dried in air. The transient photocurrent response was tested in a 1.0 M Na_2_SO_4_ aqueous solution irradiated by a 300 W xenon lamp. The electrochemical impedance spectra (EIS) were obtained over a frequency range from 0.01 to 10^5^ at an applied potential of 0.2 V using a 10 mM K_3_[Fe(CN)_6_]/K_4_[Fe(CN)_6_] aqueous solution.

### 3.6. Evaluation of Photocatalytic Activity

Photocatalytic hydrogen production was performed in a vacuum-closed gas circulation system (Lab-solar 6A, perfectlight, Beijing, China). In a typical photocatalytic process, 10 mg of each photocatalyst was dispersed into 100 mL of an aqueous solution containing 10% TEOA as a sacrificial agent. Pt of 3 wt% (theoretical amount) was loaded onto the photocatalysts as a cocatalyst by in situ photo-deposition. Before illumination, the reaction system was evacuated for 30 min to remove air, and the hydrogen yield was measured by online gas chromatography (PANNA, A91, Changzhou, China) using Ar as a carrier gas.

The apparent quantum efficiency (AQE) was measured under the same conditions. The light irradiation area was 19 cm^2^. The amount of hydrogen evolution was obtained using a 300 W Xe lamp (CEL-HXF300-T3, Beijing, China) as a light source and a 420 nm band-pass filter to allow the corresponding wavelength photons to pass through. The AQE was calculated by the following equation:

(3) $$\mathrm{AQE}\;(\%)=\frac{\mathrm{Number}\;\mathrm{of}\;\mathrm{reacted}\;\mathrm{electrons}}{\mathrm{Number}\;\mathrm{of}\;\mathrm{incident}\;\mathrm{photons}}\;\times \;100\%=\frac{{\mathrm{Number}\;\mathrm{of}\;\mathrm{evolved}\;H}_{2}\;\mathrm{molecules}\;\times \;2}{\mathrm{Number}\;\mathrm{of}\;\mathrm{incident}\;\mathrm{photons}}$$

## 4. Conclusions

In conclusion, we designed a PCNS photocatalyst with a large specific surface area through P-doping and thermal exfoliation reaction processes. The hydrogen evolution rate of 700 μmol·g^−1^·h^−1^ of the PCNS was 43.8 times higher than that of g-C_3_N_4_. Its apparent quantum efficiency reached up to 0.49%. The PCNS displayed excellent cycling and hydrogen evolution stability. Its outstanding photocatalytic performance was ascribed to its thin 2D structure and large surface area affording more reactive sites, further promoting the photogenerated hole–electron pairs’ separation and transfer. This work provides a facile and effective method to prepare PCNSs with excellent photocatalytic activity for hydrogen evolution, and this method confirms the potential of using non-metal doping and thermal exfoliation for the future optimization of high-performance solar-driven water-splitting catalysts.

## Data Availability

The data are contained within this article.

## Supplementary Materials

The following materials are available online at https://www.mdpi.com/article/10.3390/molecules29153666/s1. Figure S1: SEM images of (a) CNS and (b) PCN; Figure S2: N_2_ adsorption–desorption isotherm curves of four photocatalysts; Figure S3: PL spectra of CN and PCNS; Table S1: Comparison of the H2 evolution rates between the current work and other reports [46,47,48,49,50].

## References

[1] Wang M., Cheng J., Wang X., Hong X., Fan J., Yu H. (2021). Sulfur-mediated photodeposition synthesis of NiS cocatalyst for boosting H_2_-evolution performance of g-C_3_N_4_ photocatalyst. Chin. J. Catal..

[2] Wang R., Cao X., Huang H., Ji X., Chen X., Liu J., Yan P., Wei S., Chen L., Wang Y. (2022). Facile Chemical Vapor Modification Strategy to Construct Surface Cyano-Rich Polymer Carbon Nitrides for Highly Efficient Photocatalytic H_2_ Evolution. ChemSusChem.

[3] Wang S., Wang X., Liu B., Xiao X., Wang L., Huang W. (2022). Boosting the photocatalytic hydrogen production performance of graphitic carbon nitride nanosheets by tailoring the cyano groups. J. Colloid Interface Sci..

[4] Wang X., Jin Z. (2024). Adjusting inter-semiconductor barrier height via crystal plane engineering: Crystalline face exposed single crystal cadmium sulfide augmentative S-scheme heterojunctions for efficiently photocatalytic hydrogen production. Appl. Catal. B Environ..

[5] Zhai H., Tan P., Lu L., Liu H., Liu Y., Pan J. (2021). Abundant hydroxyl groups decorated on nitrogen vacancy-embedded g-C_3_N_4_ with efficient photocatalytic hydrogen evolution performance. Catal. Sci. Technol..

[6] Zhang Q., Sun Y., Deng J., Liu Y., Ng Y.H., Jing L., Dai H. (2023). Defect-rich selenium doped graphitic carbon nitride for high-efficiency hydrogen evolution photocatalysis. Int. J. Hydrogen Energy.

[7] Sun X.-J., Yang D.-D., Dong H., Meng X.-B., Sheng J.-L., Zhang X., Wei J.-Z., Zhang F.-M. (2018). ZIF-derived CoP as a cocatalyst for enhanced photocatalytic H_2_ production activity of g-C_3_N_4_. Sustain. Energy Fuels.

[8] Chen Z., Xia K., She X., Mo Z., Zhao S., Yi J., Xu Y., Chen H., Xu H., Li H. (2018). 1D metallic MoO_2_-C as co-catalyst on 2D g-C_3_N_4_ semiconductor to promote photocatlaytic hydrogen production. Appl. Surf. Sci..

[9] Huang Z., Chen H., He X., Fang W., Li W., Du X., Zeng X., Zhao L. (2021). Constructing a WC/NCN Schottky Junction for Rapid Electron Transfer and Enrichment for Highly Efficient Photocatalytic Hydrogen Evolution. ACS Appl. Mater. Interfaces.

[10] Deng Y., Zhou Z., Zeng H., Tang R., Li L., Wang J., Feng C., Gong D., Tang L., Huang Y. (2023). Phosphorus and kalium co-doped g-C_3_N_4_ with multiple-locus synergies to degrade atrazine: Insights into the depth analysis of the generation and role of singlet oxygen. Appl. Catal. B Environ..

[11] Fei T., Qin C., Zhang Y., Dong G., Wang Y., Zhou Y., Cui M. (2021). A 3D peony-like sulfur-doped carbon nitride synthesized by self-assembly for efficient photocatalytic hydrogen production. Int. J. Hydrogen Energy.

[12] Gong Q., Cao S., Zhou Y., Wang R., Jiao W. (2021). Mesoporous g-C_3_N_4_ decorated by Ni_2_P nanoparticles and CdS nanorods together for enhancing photocatalytic hydrogen evolution. Int. J. Hydrogen Energy.

[13] Kröger J., Jiménez-Solano A., Savasci G., Lau V.W.H., Duppel V., Moudrakovski I., Küster K., Scholz T., Gouder A., Schreiber M.L. (2021). Lotsch, Morphology Control in 2D Carbon Nitrides: Impact of Particle Size on Optoelectronic Properties and Photocatalysis. Adv. Funct. Mater..

[14] Huo Y., Zhang J., Wang Z., Dai K., Pan C., Liang C. (2021). Efficient interfacial charge transfer of 2D/2D porous carbon nitride/bismuth oxychloride step-scheme heterojunction for boosted solar-driven CO_2_ reduction. J. Colloid Interface Sci..

[15] Lei J., Gu X., Xiao P., Ding G., Yang Y., Fu X., Long B., Chen S., Meng S. (2022). Fabrication of 2D/2D BiOBr/g-C_3_N_4_ with efficient photocatalytic activity and clarification of its mechanism. Phys. Chem. Chem. Phys..

[16] Guo L., Xu F., Liu Z., Zhang M., Ma D., Lai C., Liu S., Li L., Fu Y., Qin L. (2022). Constructing benzene ring modified graphitic carbon nitride with narrowed bandgap and enhanced molecular oxygen activation for efficient photocatalytic degradation of oxytetracycline. Sep. Purif. Technol..

[17] Dang T.T., Nguyen T.K.A., Bhamu K.C., Mahvelati-Shamsabadi T., Van V.K.H., Shin E.W., Chung K.-H., Hur S.H., Choi W.M., Kang S.G. (2022). Engineering Holey Defects on 2D Graphitic Carbon Nitride Nanosheets by Solvolysis in Organic Solvents. ACS Catal..

[18] Gao S., Wang X., Song C., Zhou S., Yang F., Kong Y. (2021). Engineering carbon-defects on ultrathin g-C_3_N_4_ allows one-pot output and dramatically boosts photoredox catalytic activity. Appl. Catal. B Environ..

[19] Lei L., Wang W., Shang Y., Li J., Yadav A.K., Wang H., Li Q., Fan H. (2021). Tailoring chemical structures and intermolecular interactions of melem intermediates for highly efficient photocatalytic hydrogen evolution of g-C_3_N_4_. Appl. Surf. Sci..

[20] Shen H., Li M., Guo W., Li G., Xu C. (2020). P, K co-doped porous g-C_3_N_4_ with enhanced photocatalytic activity synthesized in vapor and self-producing NH_3_ atmosphere. Appl. Surf. Sci..

[21] Chen L., Yan G., Liu X., Ying S., Xia Y., Ning S., Wang X. (2022). Phosphorus doped and defect modified graphitic carbon nitride for boosting photocatalytic hydrogen production. Phys. Chem. Chem. Phys..

[22] Ou H., Lin L., Zheng Y., Yang P., Fang Y., Wang X. (2017). Tri-s-triazine-Based Crystalline Carbon Nitride Nanosheets for an Improved Hydrogen Evolution. Adv. Mater..

[23] Ren M., Zhang X., Liu Y., Yang G., Qin L., Meng J., Guo Y., Yang Y. (2022). Interlayer Palladium-Single-Atom-Coordinated Cyano-Group-Rich Graphitic Carbon Nitride for Enhanced Photocatalytic Hydrogen Production Performance. ACS Catal..

[24] Shen R., Xie J., Lu X., Chen X., Li X. (2018). Bifunctional Cu_3_P Decorated g-C_3_N_4_ Nanosheets as a Highly Active and Robust Visible-Light Photocatalyst for H_2_ Production. ACS Sustain. Chem. Eng..

[25] Shi W., Shu K., Sun H., Ren H., Li M., Chen F., Guo F. (2020). Dual enhancement of capturing photogenerated electrons by loading CoP nanoparticles on N-deficient graphitic carbon nitride for efficient photocatalytic degradation of tetracycline under visible light. Sep. Purif. Technol..

[26] Bai X., Wang X., Lu X., Hou S., Sun B., Wang C., Jia T., Yang S. (2021). High crystallinity and conjugation promote the polarization degree in O-doped g-C_3_N_4_ for removing organic pollutants. CrystEngComm.

[27] Beyhaqi A., Azimi S.M.T., Chen Z., Hu C., Zeng Q. (2021). Exfoliated and plicated g-C_3_N_4_ nanosheets for efficient photocatalytic organic degradation and hydrogen evolution. Int. J. Hydrogen Energy.

[28] Bi F., Su Y., Zhang Y., Chen M., Darr J.A., Weng X., Wu Z. (2022). Vacancy-defect semiconductor quantum dots induced an S-scheme charge transfer pathway in 0D/2D structures under visible-light irradiation. Appl. Catal. B Environ..

[29] Chen H., Wang W., Yang Z., Suo X., Lu Z., Xiao W., Dai S. (2021). Alkaline salt-promoted construction of hydrophilic and nitrogen deficient graphitic carbon nitride with highly improved photocatalytic efficiency. J. Mater. Chem. A.

[30] Chen J., Zhu X., Jiang Z., Zhang W., Ji H., Zhu X., Song Y., Mo Z., Li H., Xu H. (2022). Construction of brown mesoporous carbon nitride with a wide spectral response for high performance photocatalytic H_2_ evolution. Inorg. Chem. Front..

[31] Chen Y., Lei L., Gong Y., Wang H., Fan H., Wang W. (2023). Enhanced electron delocalization on pyrimidine doped graphitic carbon nitride for boosting photocatalytic hydrogen evolution. Int. J. Hydrogen Energy.

[32] Cheng J., Hou Y., Lian K., Xiao H., Lin S., Wang X. (2022). Metalized Carbon Nitrides for Efficient Catalytic Functionalization of CO_2_. ACS Catal..

[33] Song T., Zhang X., Yang P. (2022). Interface engineering of W_2_C/W_2_N co-catalyst on g-C_3_N_4_ nanosheets for boosted H_2_ evolution and 4-nitrophenol removal. Environ. Sci. Nano.

[34] Li P., Wang M., Huang S., Su Y. (2021). Phosphorus- and fluorine-co-doped carbon nitride: Modulated visible light absorption, charge carrier kinetics and boosted photocatalytic hydrogen evolution. Dalton Trans..

[35] Ma J., Jin D., Yang X., Sun S., Zhou J., Sun R. (2021). Phosphorus-doped carbon nitride with grafted sulfonic acid groups for efficient photocatalytic synthesis of xylonic acid. Green Chem..

[36] Sun K., Shen J., Liu Q., Tang H., Zhang M., Zulfiqar S., Lei C. (2020). Synergistic effect of Co(II)-hole and Pt-electron cocatalysts for enhanced photocatalytic hydrogen evolution performance of P-doped g-C_3_N_4_. Chin. J. Catal..

[37] Xu J., Qi Y., Wang W., Wang L. (2019). Montmorillonite-hybridized g-C_3_N_4_ composite modified by NiCoP cocatalyst for efficient visible-light-driven photocatalytic hydrogen evolution by dye-sensitization. Int. J. Hydrogen Energy.

[38] Liu F., Li W., Wang L., Rao X., Zheng S., Zhang Y. (2022). Sulfur- and Strontium-Doped Graphitic Carbon Nitride for Efficient Photocatalytic Hydrogen Evolution. ACS Appl. Energy Mater..

[39] Liu W., Peng R., Ye X., Guo J., Luo L. (2021). Sulfur doping and structure defect functionalized carbon nitride nanosheets with enhanced photocatalytic degradation activity. Appl. Surf. Sci..

[40] Luo L., Wang K., Gong Z., Zhu H., Ma J., Xiong L., Tang J. (2021). Bridging-nitrogen defects modified graphitic carbon nitride nanosheet for boosted photocatalytic hydrogen production. Int. J. Hydrogen Energy.

[41] Wang D., Dong X., Lei Y., Lin C., Huang D., Yu X., Zhang X. (2022). Fabrication of Mn/P co-doped hollow tubular carbon nitride by a one-step hydrothermal–calcination method for the photocatalytic degradation of organic pollutants. Catal. Sci. Technol..

[42] Liu W., Zhang D., Wang R., Zhang Z., Qiu S. (2022). 2D/2D Interface Engineering Promotes Charge Separation of Mo_2_C/g-C_3_N_4_ Nanojunction Photocatalysts for Efficient Photocatalytic Hydrogen Evolution. ACS Appl. Mater. Interfaces.

[43] Liu Y., Zhao S., Wang Y., Xie L., Fang J., Zhang Y., Zhou Y., Zhuo S. (2021). Controllable fabrication of 3D porous carbon nitride with ultra-thin nanosheets templated by ionic liquid for highly efficient water splitting. Int. J. Hydrogen Energy.

[44] Long X., Feng C., Yang S., Ding D., Feng J., Liu M., Chen Y., Tan J., Peng X., Shi J. (2022). Oxygen doped graphitic carbon nitride with regulatable local electron density and band structure for improved photocatalytic degradation of bisphenol A. Chem. Eng. J..

[45] Pan Z., Liu M., Zhang G., Zhuzhang H., Wang X. (2021). Molecular Triazine–Heptazine Junctions Promoting Exciton Dissociation for Overall Water Splitting with Visible Light. J. Phys. Chem. C.

[46] Zhou Y., Lv W., Zhu B., Tong F., Pan J., Bai J., Zhou Q., Qin H. (2019). Template-Free One-Step Synthesis of g-C_3_N_4_ Nanosheets with Simultaneous Porous Network and S-Doping for Remarkable Visible-Light-Driven Hydrogen Evolution. ACS. Sustain. Chem. Eng..

[47] Chava R.K., Kang M. (2023). Ordered and carbon-doped porous polymeric graphitic carbon nitride nanosheets toward enhanced visible light absorption and efficient photocatalytic H_2_ evolution. Nanoscale.

[48] Guo S., Deng Z., Li M., Jiang B., Tian C., Pan Q., Fu H. (2015). Phosphorus-Doped Carbon Nitride Tubes with a Layered Micro-nanostructure for Enhanced Visible-Light Photocatalytic Hydrogen Evolution. Angew. Chem..

[49] Guo S., Tang Y., Xie Y., Tian C., Feng Q., Zhou W., Jiang B. (2017). P-doped tubular g-C3N4 with surface carbon defects: Universal synthesis and enhanced visible-light photocatalytic hydrogen production. Appl. Catal. B Environ..

[50] Zhou Y., Zhang L., Liu J., Fan X., Wang B., Wang M., Ren W., Wang J., Li M., Shi J. (2015). Brand new P-doped g-C_3_N_4_: Enhanced photocatalytic activity for H_2_ evolution and Rhodamine B degradation under visible light. J. Mater. Chem. A.

