# Supplementary material for: Thermal Exfoliation and Phosphorus Doping in Graphitic Carbon Nitride for Efficient Photocatalytic Hydrogen Production
Source: Molecules. 2024 Aug 2;29(15):3666. doi: 10.3390/molecules29153666 (PMC11314274; doi:10.3390/molecules29153666)
Supplement: Supplementary file 1 [file molecules-29-03666-s001.zip › molecules-3104216-supplementary.pdf]

Thermal exfoliation and phosphorus doping in graphitic carbon nitride for efficient photocatalytic hydrogen production

Lu Chen[1]*, Linzhu Zhang[1], Yuzhou Xia[1], Renkun Huang[1], Ruowen Liang[1], Guiyang Yan[1*], Xuxu Wang[1]*

[1]Fujian Province University Key Laboratory of Green Energy and Environment Catalysis, Ningde Normal University, Ningde 352100, PR China

[2] State Key Laboratory of Photocatalysis on Energy and Environment, Fuzhou University, Fuzhou 350002, P. R. China

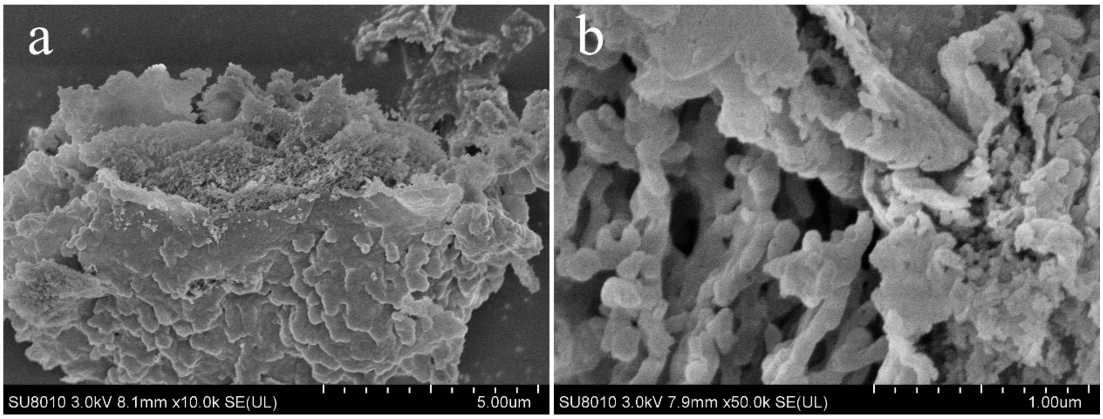

Fig. S1 SEM images of (a) CNS, (b) PCN

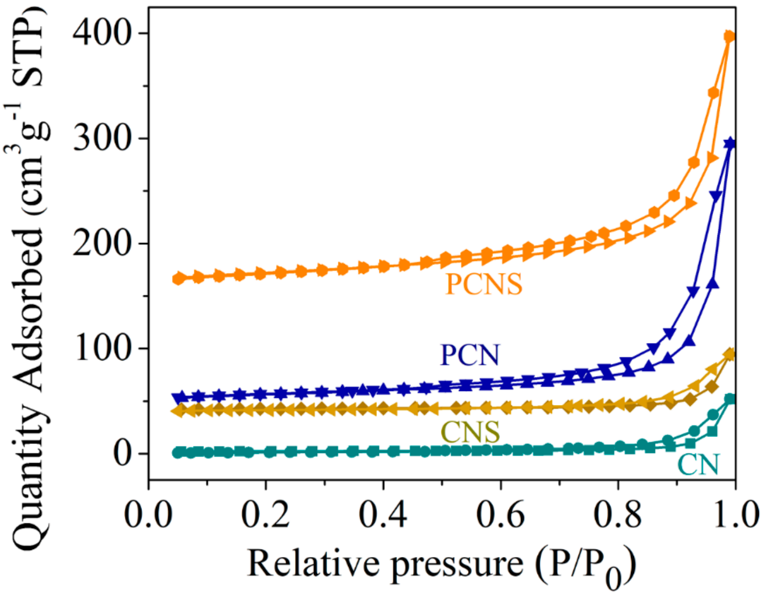

Fig. S2 N_2 adsorption–desorption isotherm curves of four photocatalysts

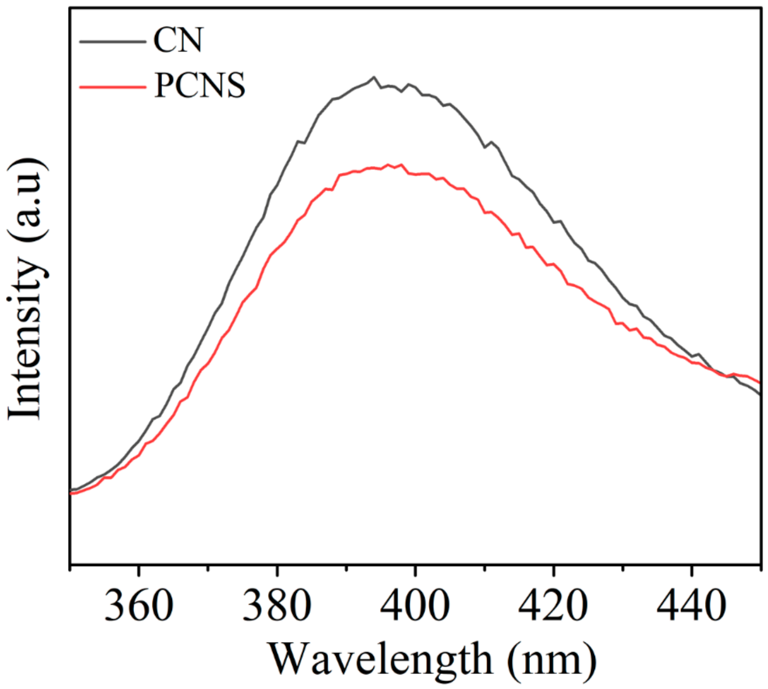

Fig. S3 PL spectra of CN and PCNS

Table. S1 Comparison of the H_2 evolution rates between the current work and other reports

Sample	Condition	Weight (mg)	H_2 (ummol·g^{-1}·h^{-1})	Ref
P-g-C_3N_4	10% TEOA (λ> 420 nm)	30	700	Our work
S-g-C_3N_4	10% TEOA (λ> 420 nm)	50	142	1
S-g-C_3N_4	10% TEOA (λ> 420 nm)	15	367	2
C-g-C_3N_4	10% TEOA (λ> 420 nm)	25	663.6	3
P-g-C_3N_4	10% TEOA (λ> 420 nm)	100	670	4
P-g-C_3N_4	10% TEOA (λ> 420 nm)	100	570	5
P-g-C_3N_4	10% TEOA (λ> 420 nm)	100	506	6

reference

[1] T. Fei, C. Qin, Y. Zhang, G. Dong, Y. Wang, Y. Zhou, M. Cui, A 3D peony-like sulfur-doped carbon nitride synthesized by self-assembly for efficient photocatalytic hydrogen production, International Journal of Hydrogen Energy, 46 (2021) 20481-

20491.
[2] Y. Zhou, W. Lv, B. Zhu, F. Tong, J. Pan, J. Bai, Q. Zhou, H. Qin, Template-Free One-Step Synthesis of g-C_3N_4 Nanosheets with Simultaneous Porous Network and S-Doping for Remarkable Visible-Light-Driven Hydrogen Evolution, ACS Sustainable Chemistry & Engineering, 7 (2019) 5801-5807.
[3] R.K. Chava, M. Kang, Ordered and carbon-doped porous polymeric graphitic carbon nitride nanosheets toward enhanced visible light absorption and efficient photocatalytic H_2 evolution, Nanoscale, 15 (2023) 18347-18358.
[4] S. Guo, Z. Deng, M. Li, B. Jiang, C. Tian, Q. Pan, H. Fu, Phosphorus-Doped Carbon Nitride Tubes with a Layered Micro-nanostructure for Enhanced Visible-Light Photocatalytic Hydrogen Evolution, Angewandte Chemie International Edition, 55 (2015) 1830-1834.
[5] S. Guo, Y. Tang, Y. Xie, C. Tian, Q. Feng, W. Zhou, B. Jiang, P-doped tubular g-C3N4 with surface carbon defects: Universal synthesis and enhanced visible-light photocatalytic hydrogen production, Applied Catalysis B: Environmental, 218 (2017) 664-671.
[6] Y. Zhou, L. Zhang, J. Liu, X. Fan, B. Wang, M. Wang, W. Ren, J. Wang, M. Li, J. Shi, Brand new P-doped g-C_3N_4: enhanced photocatalytic activity for H_2 evolution and Rhodamine B degradation under visible light, Journal of Materials Chemistry A, 3 (2015) 3862-3867.
